# Two Cases—One of Gouty Inflammation of the Testis, and Another the Testicle of Mumps

**Published:** 1883-12

**Authors:** F. K. Green

**Affiliations:** Surgeon Bath Mineral Water Hospital, and Senior Assistant Surgeon Royal United Hospital


### TWO CASES—ONE OF GOUTY INFLAMMATION OF THE TESTIS, AND ANOTHER THE TESTICLE OF MUMPS. 

By F. K. Green, F.R.C.S., Surgeon Bath Mineral Water Hospital, and Senior Assistant Surgeon Royal United Hospital.

The two cases here given were of interest to me, occurring as they did about the same time and bearing directly upon the question of metastasis.

### Case of Gouty Inflammation of the Testis.

E. G., æt. 52, married, was a cachectic, broken-down hotel porter. When I was requested to see him I was told that he was suffering from orchitis, the result of injury, and that there was probably a hæmatocele. To begin with I was thrown off my guard.

A little inquiry convinced me that the supposed injury was in reality not the cause of his trouble, and examination showed that no hæmatocele existed. I suspected gonorrhœa, which (the patient being married) was, of course, denied. I found acute epididymitis, also inflammation of the gland proper; slight effusion into the tunica vaginalis (which with transmitted light I judged to be turbid), as well as into the posterior part of scrotum; some thickening of the vas and effusion into the cord; exquisite tenderness.

I ordered a saline, with tartar emetic, hot fomentations and bed.

On the sixth day of treatment no improvement had resulted. On the evening of that day he had a sharp attack of orthopnœa, with severe pain in right mammary region, the initial symptoms of a pneumonia which pursued a mild course. On the twelfth day occurred an effusion into both knee-joints, and at the same time there was a slight diminution in the pain in the testicle.

With such symptoms I inquired further into the patient's history, and found that he had suffered from numerous attacks of gouty arthritis. I now inclined to the opinion that the orchitis was gouty, and the further progress of the case confirmed this opinion. Iodide of potassium and colchicum were ordered.

At the end of a further four days but little progress had been made in the testicle and knee-joints, although the pneumonic symptoms were subsiding; but at this date he had a nocturnal seizure of gout in the right foot, which, in the morning, from the ball of the great toe to the ankle was swollen and of a dusky-red colour, and great was my pleasure to find that the pain in the testicle was entirely gone and the swelling considerably diminished. The rapid recovery of the testicle dated from this period, as also that of the arthritic effusions. Not so, however, the gout in the foot. Several weeks elapsed before the patient could leave his bed, and many more before he could put on his boot.

After the acute symptoms had subsided I made several examinations of his urine and found a low sp. gr. of 1010, and although it contained no albumen, yet the presence here and there of a faintly granular cast revealed the true state of his kidneys.

### Case 2.—The Testicle of Mumps.

J. H., æt. 35, a married man, healthy and well-built, was seized in the night with violent delirium, which had somewhat subsided when I saw him on the following morning, although great restlessness was still present. I found well-marked pyrexia; T., 104°·6; pulse, 132; slight commencing perspiration; scanty high-coloured urine. Examination revealed no chest symptoms, no sign of any of the exanthemata, but I accidentally found the left testicle greatly enlarged, hard, and excessively tender. The epididymis shared in this condition, although the gland-swelling greatly preponderated. The shape of the organ was more globular than is invariably seen in gonorrhœal epididymitis, in which, as a rule, there is some flattening from side to side; neither was there the marked prominence of the globus minor, so characteristic of the latter affection. There was slight effusion into the cord, but the vas was not perceptibly thickened.

From the patient in the condition he was in I could of course gain no history of urethral discharge, and I was loth to question the wife on such a matter; but on asking if he had looked ill for some days past I elicited that fourteen or sixteen days before he had had a great swelling behind each angle of the jaw, which in a few days had completely subsided, and on now examining the parotid regions I could detect no tangible swelling or harshness.

The connection of the orchitis with mumps seemed fairly made out. Acetate of ammonia and antimony were prescribed, together with hot fomentations to the testicle. During the following night there was again delirium, but in the morning his temperature had fallen, and on the fifth day he was convalescent, although very weak. The pain and swelling of the testis rapidly subsided.

*Remarks.*—The foregoing cases suggest two questions: First, Have the gouty testicle and the testicle of mumps any characters by which they may he distinguished from the common affection gonorrhœal epididymitis? In the case of gout I could detect no appearance in the testicle which is not present in the gonorrhœal affection. The stress of inflammation was in the epididymis. Its slowness to yield to antimonial salines might perhaps have suggested its non-venereal origin; on the other hand, gonorrhœal epididymitis is sometimes very slow in resolution. A history of urethral discharge helps us not at all, for that may be a distinctly, although rare, gouty affection. Indeed, there are some who believe that the gouty testicle, like the gonorrhœal, is always preceded by urethritis. I fear that nothing but a clear history of gout can avail us in diagnosis.

In the case of mumps the globular appearance of the swelling was noticeable, so different from the flattening from side to side seen in gonorrhœa. Again, the stress of inflammation was in the testicle, or gland proper, while the epididymis was in a minor degree enlarged, the reverse of what obtains in the venereal affection.

The second question suggested by these cases is the vexed one of metastasis. What is metastasis? Can any one answer?

"Modern pathology, whilst admitting the existence of the phenomena to which the term metastasis has been applied, refuses to accept as satisfactory the explanation of the fact implied in the term."*

This is all I can gather of metastasis in the most recent *Dictionary of Medicine.*

The long-prevalent opinion that orchitis is usually a sympathetic inflammation has given place recently to the theory of extension of inflammation along the vas deferens from the urethra to the testicle; and doubtless this is true of gonorrhœal epididymitis, and possibly may also happen in gout when urethritis, a symptom not altogether rare in gout, exists. But gouty orchitis may exist without any urethritis, and I am not aware that urethral symptoms are recognised as part of the disease called mumps. Yet there are some advocates of this theory, Kocher in particular, who believe that urethritis always exists. Kocher believes that the parotitis of mumps is always preceded by stomatitis, which is propagated through Steno's duct to the salivary gland, and that the poison of mumps in its elimination by the kidneys sets up a cystitis or urethritis, which subsequently travelling through the vas reaches at last the testicle. This is ingenious in theory, but I do not think it is borne out in practice. The advocates of this theory ignore metastasis to the testicle in gout and mumps. I would ask, how do they account for inflamma-

* Quain's *Dictionary of Medicine.*

tion of the mamma in mumps? or how account for the sudden resolution of a gouty testis, occurring simultaneously with an explosion of gout in the foot, as happened in the case before us?

Metastasis may be difficult in the present state of our knowledge to explain, although it has a certain covert meaning, but it is a convenient term, and until a better explanation is given of the cause of gout and mumps attacking the testicle, it would be well, I think, to continue to adhere to it.

